# Supplementary material for: Binding of HMGN proteins to cell specific enhancers stabilizes cell identity
Source: Nat Commun. 2018 Dec 7;9:5240. doi: 10.1038/s41467-018-07687-9 (PMC6286339; doi:10.1038/s41467-018-07687-9)
Supplement: Supplementary file 1 — Supplementary Information [file 41467_2018_7687_MOESM1_ESM.pdf]

Supplemental Information:

6 figures and figure legends, 2 Tables

Binding of HMGN Proteins to Cell Specific Enhancers Stabilizes Cell Identity

Bing He [1], Tao Deng[1#], Iris Zhu[2#], Takashi Furusawa[1], Shaofei Zhang[1], Wei Tang[3], Yuri Postnikov[1], Stefan Ambs[3], Caiyi Cherry Li [4], Ferenc Livak [4], David Landsman[2] and Michael Bustin[1*]

[1]Protein Section, Laboratory of Metabolism, Center for Cancer Research, National Cancer Institute, [2] Computational Biology Branch, National Center for Biotechnology Information, National Library of Medicine, [3]Laboratory of Human Carcinogenesis, Center for Cancer Research, [4]Laboratory of Genomic Integrity, Center for Cancer Research National Cancer Institute National Institutes of Health, Bethesda, MD 20892

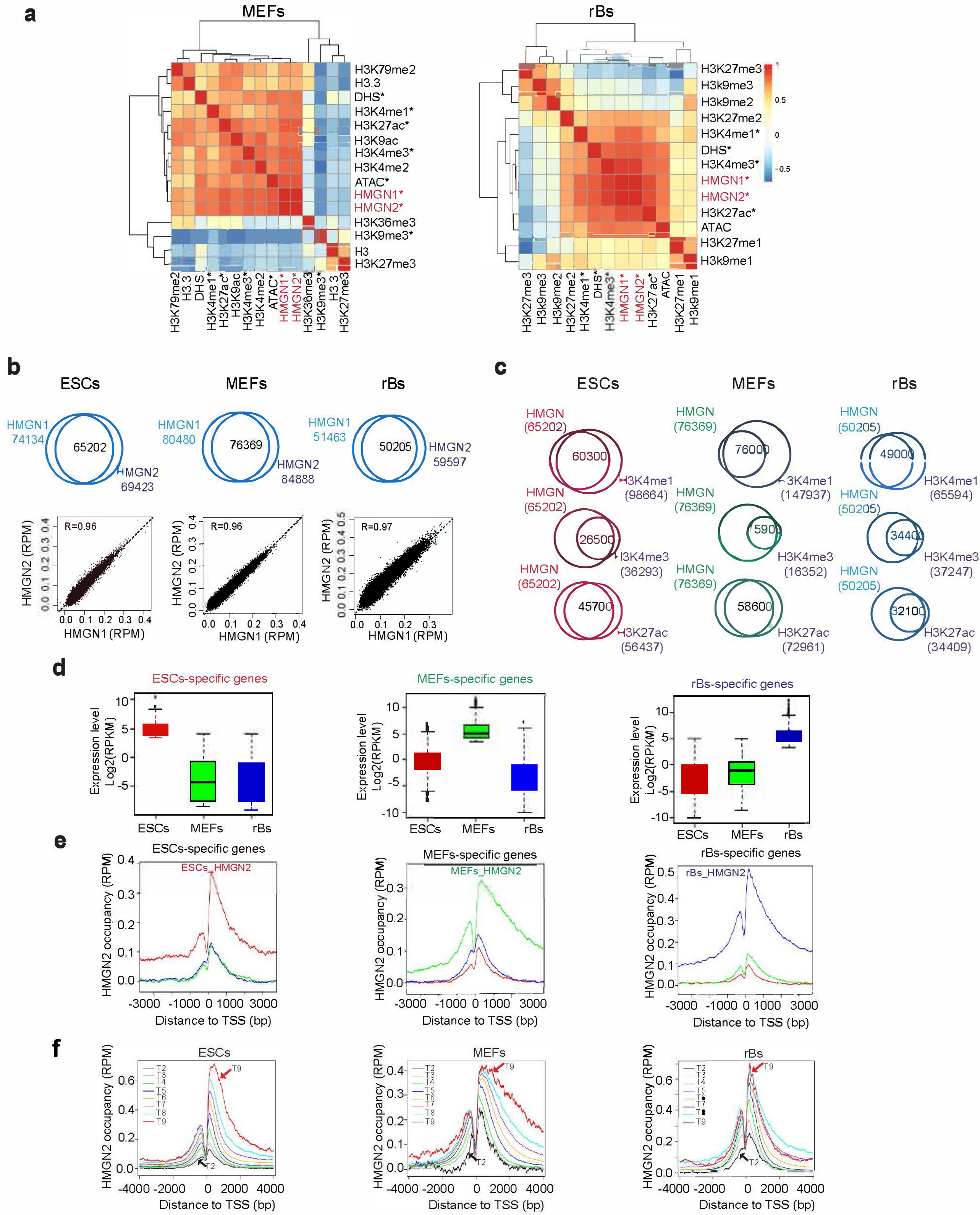

Supplementary Figure 1. HMGNs localize to tissue specific chromatin regulatory sites.
a, Clustering map showing genome wide preferential localization of HMGN1 and HMGN2 in rBs and MEFs, with epigenetic marks of active, but not of inactive chromatin. *data from our laboratory. **b,** Genome wide colocalization of HMGN1 and HMGN2 variants in ESCs, MEFs and rBs. **c,** Overlap of HMGN1 and HMGN2 occupancy in ESC, MEFs, and rBs with the indicated histone modifications. For this panels, the reads of HMGN1 and HMGN2 were combined. **d,** Selection of gene subsets expressed with high tissue specificity that were used for the analysis shown in Figure 1C and S1E. **e,** High HMGN2 occupancy at cell-specific expressed genes. **f,** HMGN2 occupancy at the promoter of each group sorted by expression levels shown in Fig. 1**d**.

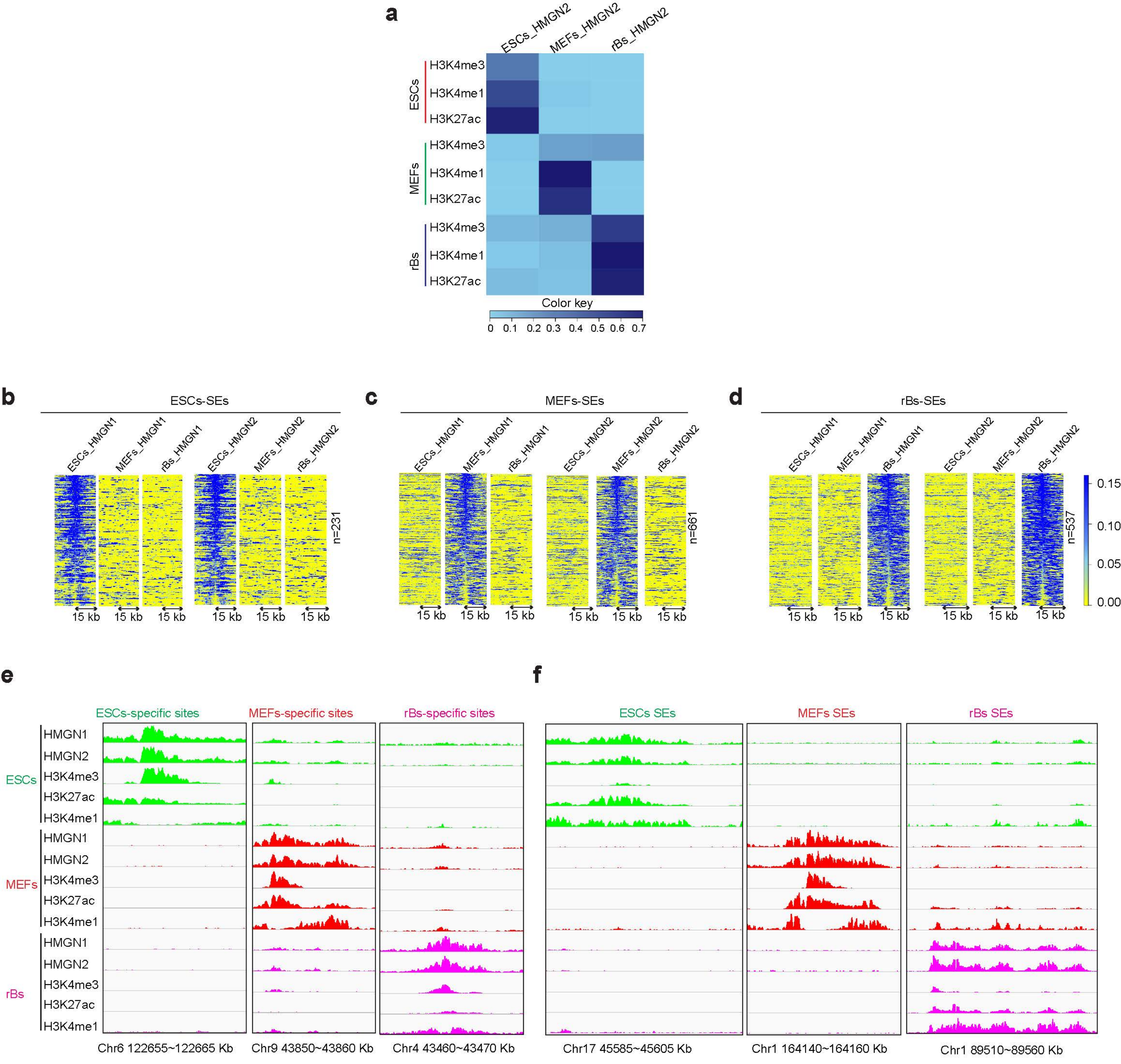

Supplementary Figure 2. **HMGNs localize to tissue specific chromatin regulatory sites.**
a, Cell type specific co-localization of HMGN2 with histone modifications marking cell type specific chromatin regulatory regions. **b-d,** Heat maps showing HMGN1 and HMGN2 clustering at cell type specific enhancer and super-enhancers of ESCs, MEFs and rBs. **e-f**, Genome browser view of the tissue specific co-occupancy of HMGN variants with chromatin regulatory sites. SE, super-enhancers

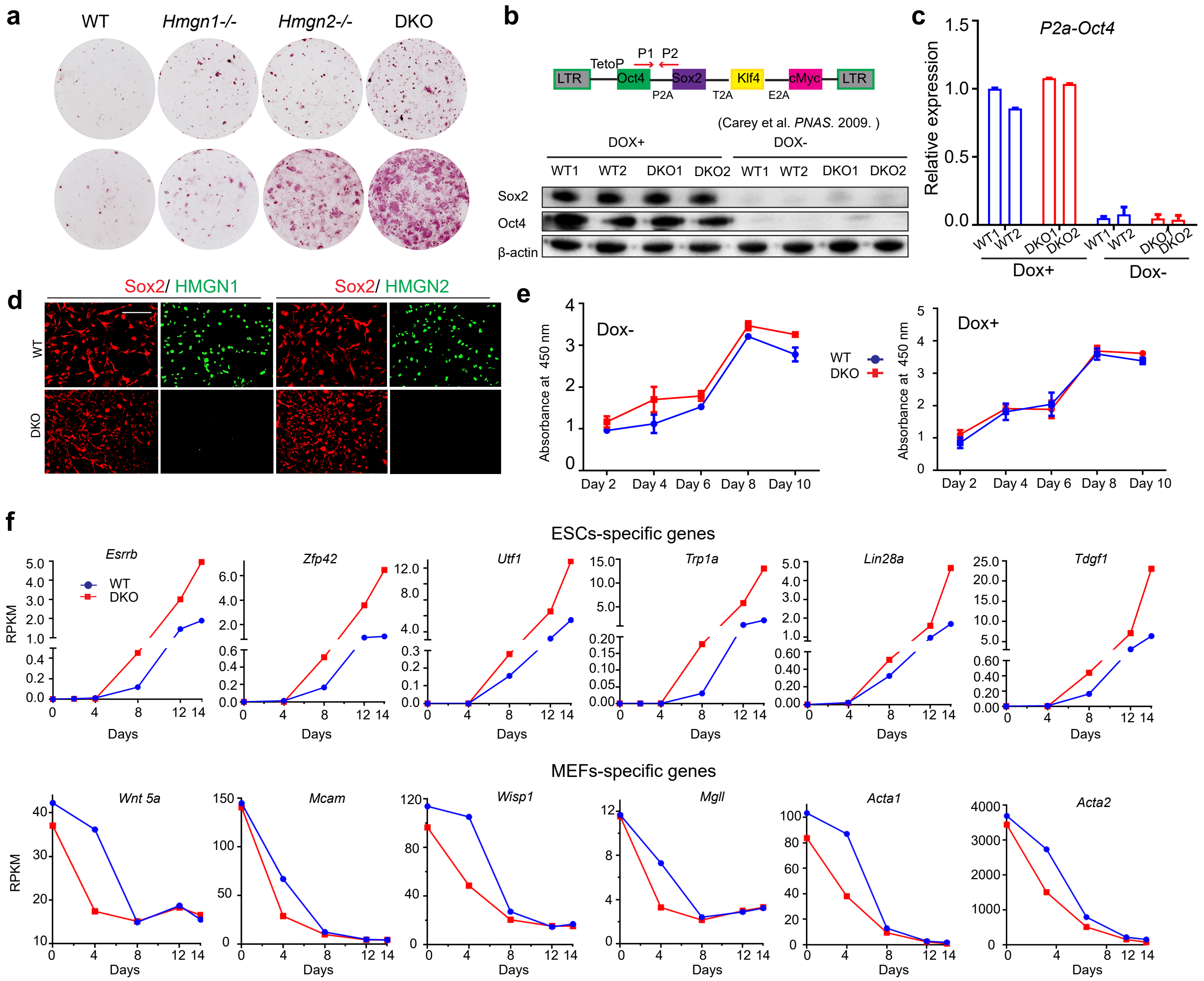

Supplementary Figure 3. Loss of HMGNs enhances the efficiency of reprogramming MEFs into iPSCs

a, Alkaline phosphatase signals in MEFs 8 days following doxycycline (DOX) induced OSKM expression. Top row: after 8 days growing in the presence of DOX. Bottom row: 8 days in the presence of DOX and 5 days without DOX. **b**, Top: map of vector used for OSKM expression. Bottom: Western analysis showing equal expression of SOX2 and OCT4 in DOX treated WT and DKO MEFs. **c,** qPCR analysis showing equal expression of lentivirus expressing OSKM in WT and DKO MEFs. The *P2A-Oct* 4 segment was amplified using primers P1 and P2. **d**, Immunofluorescence visualizing SOX2 and HMGN expression in WT and DKO MEFs 2 days following OSKM induction. **e,** Equal cells count of WT and DKO MEFs following OSKM induction, indicating equal rate of proliferation of WT and DKO cells. **f**, Expression of selected MEF- or ESC-specific genes following DOX induction of OSKM expression in WT and DKO MEFs. Note the enhanced rate of downregulation of MEF-specific genes, and upregulation of ESC-specific genes in DKO MEFs. Bar in panel c is 200µm.

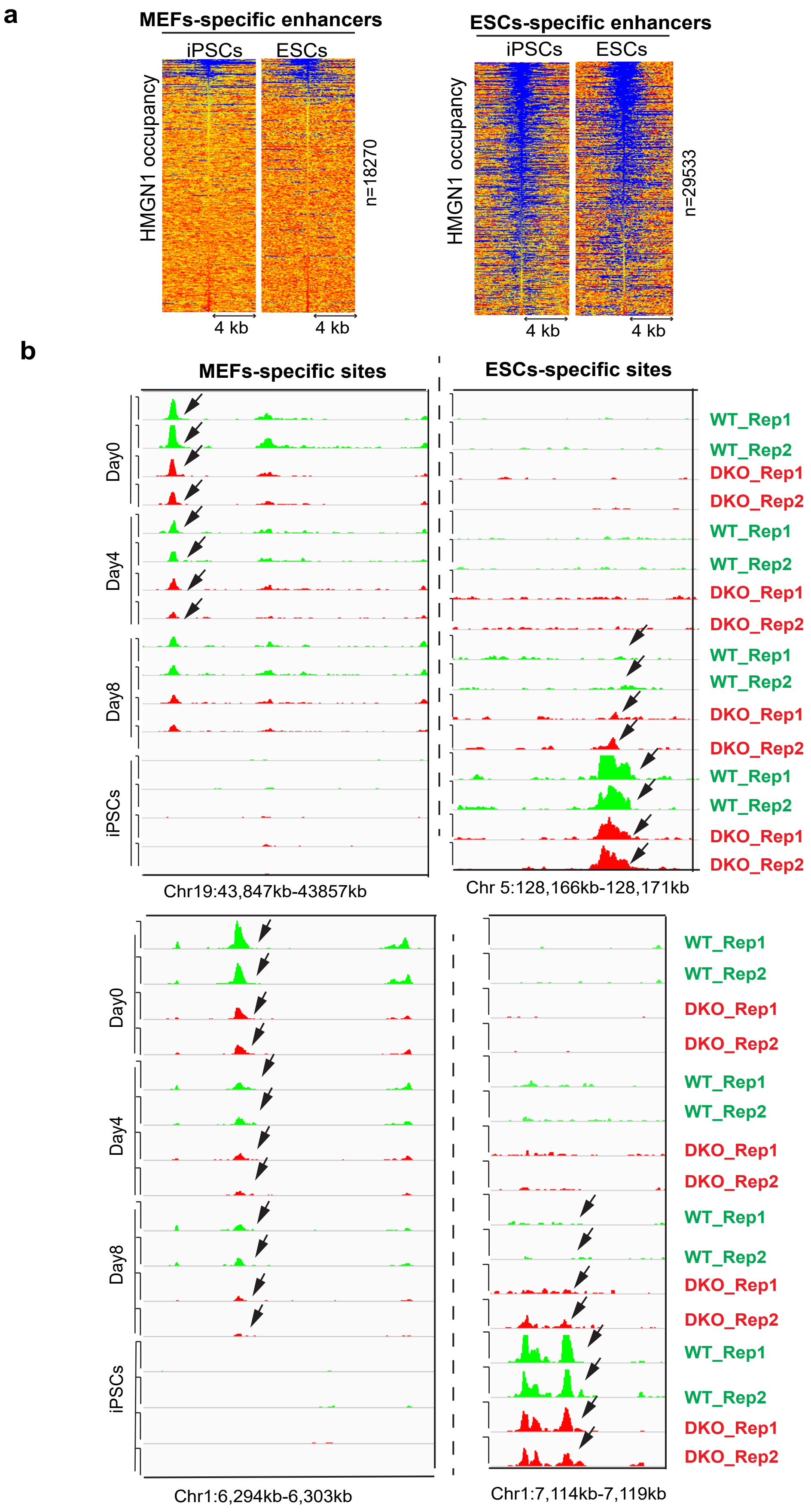

Supplementary Figure 4. HMGN1 binding at the ESC-specific and MEF-specific enhancers in ESCs and in iPSCs and ATAC dynamics during reprograming.
a, Heat map showing similarity of HMGN1 organization at the ESC-specific and MEF-specific enhancers in ESCs and in iPSCs. **b**, Genome browser visualizing the changes in ATAC sensitivity at two MEFs specific sites and two ESCs specific sites during reprograming of WT and DKO MEFs.

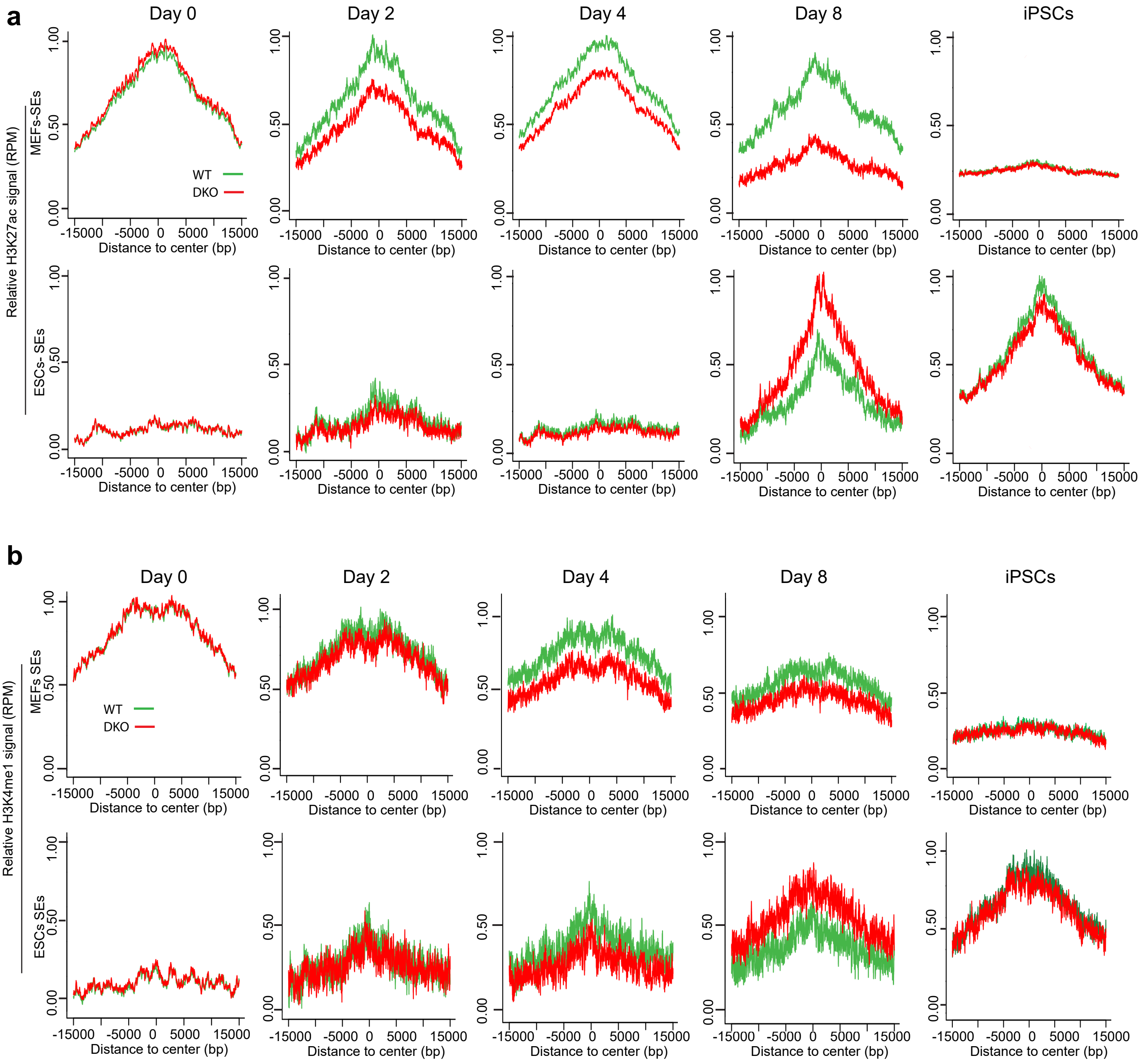

Supplementary Figure 5. Enhanced rate of epigenetic reprograming in DKO MEFs.
a, Top row: Enhanced rate of loss of H3K27ac signals at MEF super-enhancer regions in DKO cells. Bottom row: Enhanced gain of H3K27ac at ESC super-enhancers of DKO cells. **b**, Top row: Enhanced rate of loss of H3K4me1 signals at MEF super-enhancer regions in DKO cells. Bottom row: Enhanced gain of H3K4me1 at ESCs super-enhancers of DKO cells.

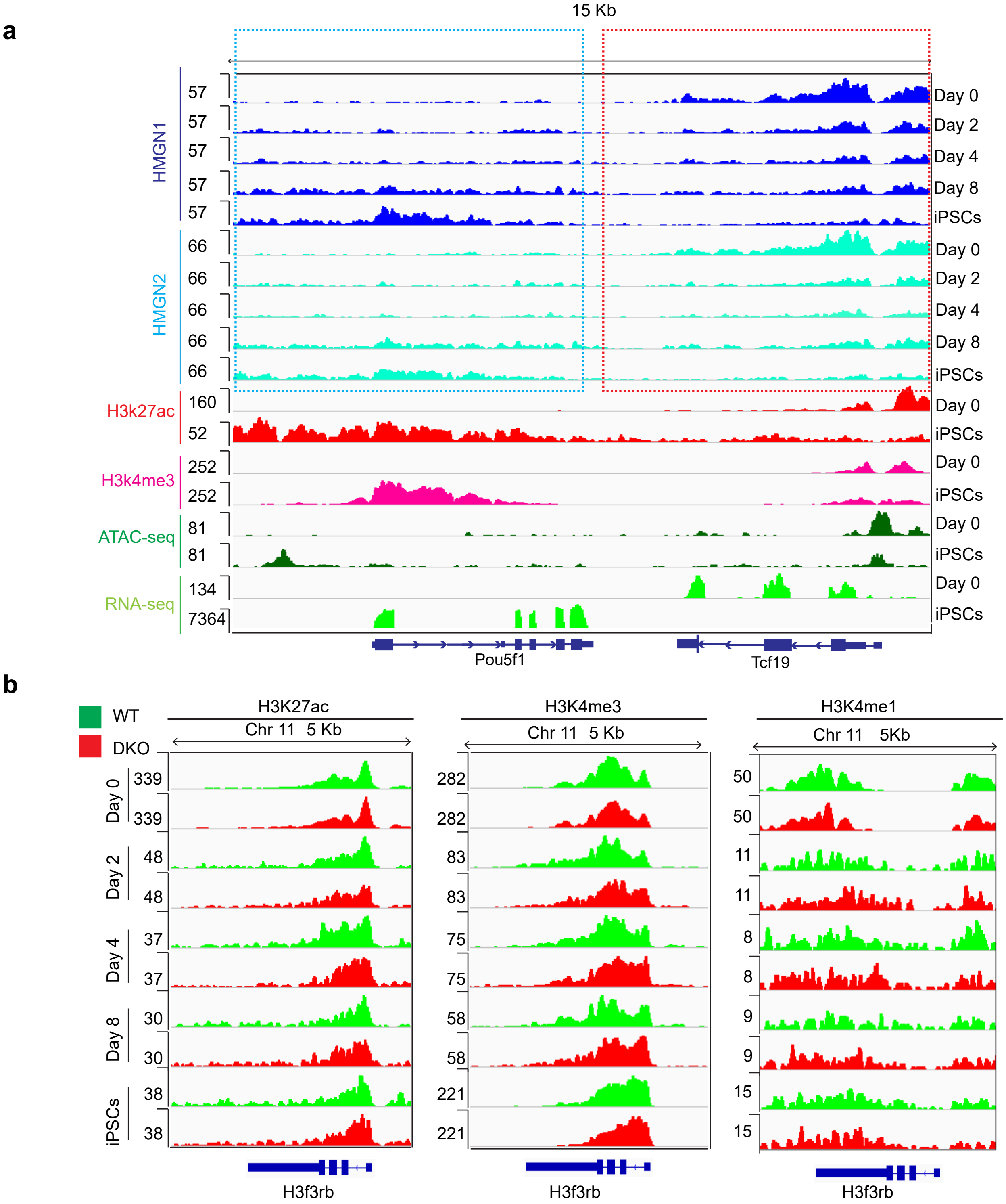

Supplementary Figure 6. HMGNs switch binding sites during reprogramming.
a, Genome browser visualizing the epigenetic changes, and the relocation of HMGNs, at two adjacent genes that are expressed either only in MEFs (*Tcf19*) or only in iPSCs (*Pou5f1*). **b,** Genome browser visualizing stable histone marks at the *H3f3rb* housekeeping gene during reprograming.

Uncropped Gels for **Fig 2 d l**eft side

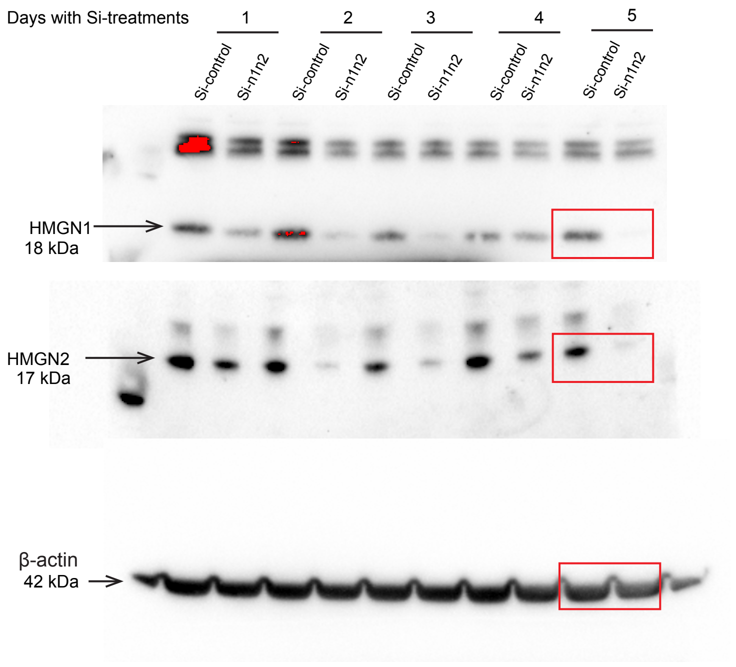

Uncropped Gels for **Fig 2 d right** side

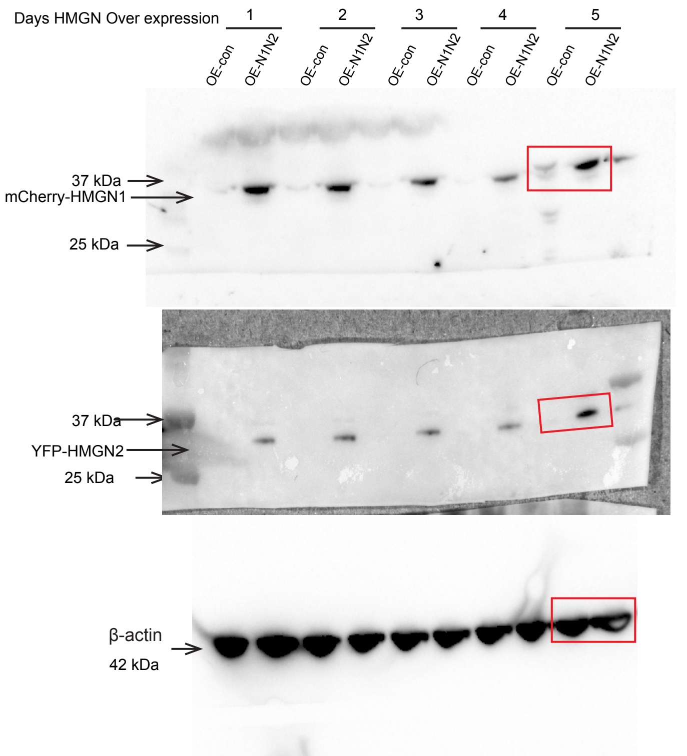

Uncropped Western blot of Fig. 6b

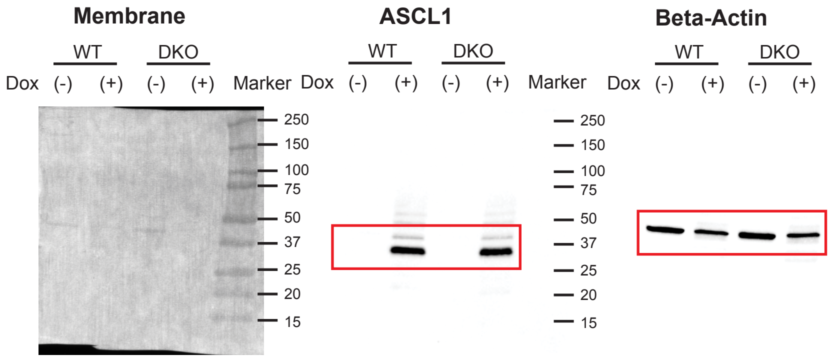

Uncropped Western blot of Supplementary Fig. 3b

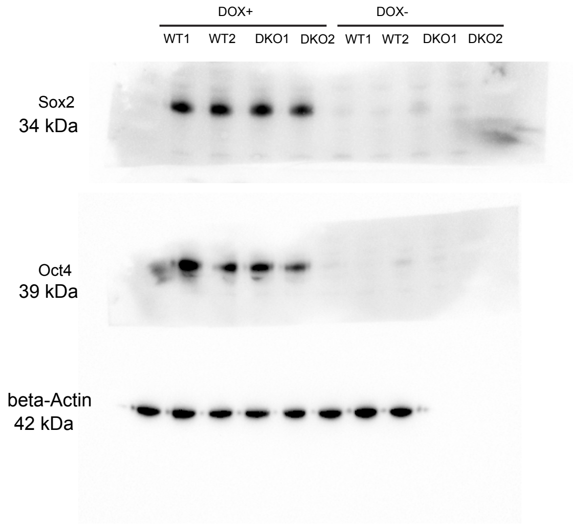

Uncropped Western blot of Fig. 6f

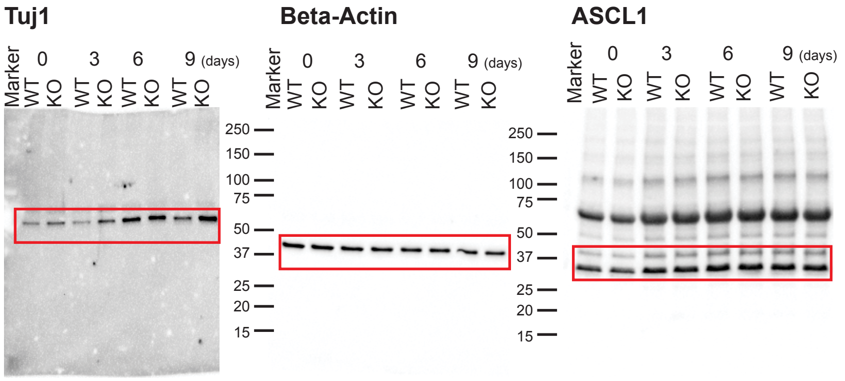

Supplementary Figure 7 . Images of uncropped gels shown in Figure 2d, Figure 6b and 6f, and in Supplementary Figure 3b.

Supplementary Table 1 qRT-PCR primers list	
**Target genes**	**Primer sequence**
P2a-Oct4_Forward	GAGGCCTTTCCCTCTGTTCC
P2a-Oct4_Reverse	ACAGAGAGAAGTTCGTGGCG
Mus_ascl1_Forward	GTCAACCTGGGTTTTGCCAC
Mus_ascl1_Reverse	TCGTTGGAGTAGTTGGGGGA
Mus_TUBB3_Forward	TGAGGCCTCCTCTCACAAGT
Mus_TUBB3_Reverse	GTCGGGCCTGAATAGGTGTC
Mus_nestin_Forward	TCGGGAGAGTCGCTTAGAGG
Mus_nestin_Reverse	CACAGCCAGCTGGAACTTTTC
Mus_Map2_Forward	CGGAAAACCACAGCAGCAAG
Mus_Map2_Reverse	GGGAGGATGGAGGAAGGTCT
Mus_Gapdh_Forward	GGAGCGAGACCCCACTAACATC
Mus_Gapdh_Reverse	CTCGTGGTTCACACCCATCAC

Supplementary Table 2 siRNA sequences for gene knockdown	
L-054969-00-0005, ON-TARGETplus Mouse *Hmgn1* (Dharmacon)	
L-062947-00-0005, ON-TARGETplus Mouse Hmgn2 (Dharmacon)	
**Target Gene**	**siRNA sequence**
Mus-Hmgn1	GAGGAAGGUUAGCGCGGAU
Mus-Hmgn1	GACUAAGCAUUCAUCACGU
Mus-Hmgn1	GGCGUAACAUUGUGAAUUG
Mus-Hmgn1	GUAGAACCAUUUUGUAAGA
Mus-Hmgn2	GAUUGAUAAUUCUGCCUAA
Mus-Hmgn2	UAAUAAAGCUGGUUACAGU
Mus-Hmgn2	UUGGUAGUCUAUACAAGAA
Mus-Hmgn2	CCUGAAAUACCAUGAUUGU
